# Supplementary material for: Unravelling the in vivo traits of vasculogenic mimicry
Source: Theranostics. 2026 Aug 12;16(15):8633–49. doi: 10.7150/thno.140146 (PMC13549142; doi:10.7150/thno.140146)
Supplement: Supplementary file 1 [file thnov16p8633s1.pdf]

Supplementary Material

Unravelling the *in vivo* traits of vasculogenic mimicry

Mariam-Eleni Oraiopoulou[1,2,*], Thierry L. Lefebvre[1,2], Eleftheria Tzamali[3], Thomas R. Else[2,1], Ian G. Cannell[1], Lorna C. Wright[1,2], Ellie V. Bunce[1,2], Cara Brodie[1], Paul W. Sweeney[1,2], Luca Porcu[1], Dominique-Laurent Couturier[4], Vangelis Sakkalis[3], Gregory J. Hannon[1, †], Sarah E. Bohndiek[1,2,*]

1 Cancer Research UK Cambridge Institute (CRUK CI), University of Cambridge, Cambridge, UK

2 Department of Physics, University of Cambridge, Cambridge, UK

3 Institute of Computer Science, Foundation for Research and Technology – Hellas, Heraklion, Greece

4 Medical Research Council Biostatistics Unit, University of Cambridge, Cambridge, UK

† Deceased during the revision of this manuscript

* Correspondence to meo29@cam.ac.uk and seb53@cam.ac.uk

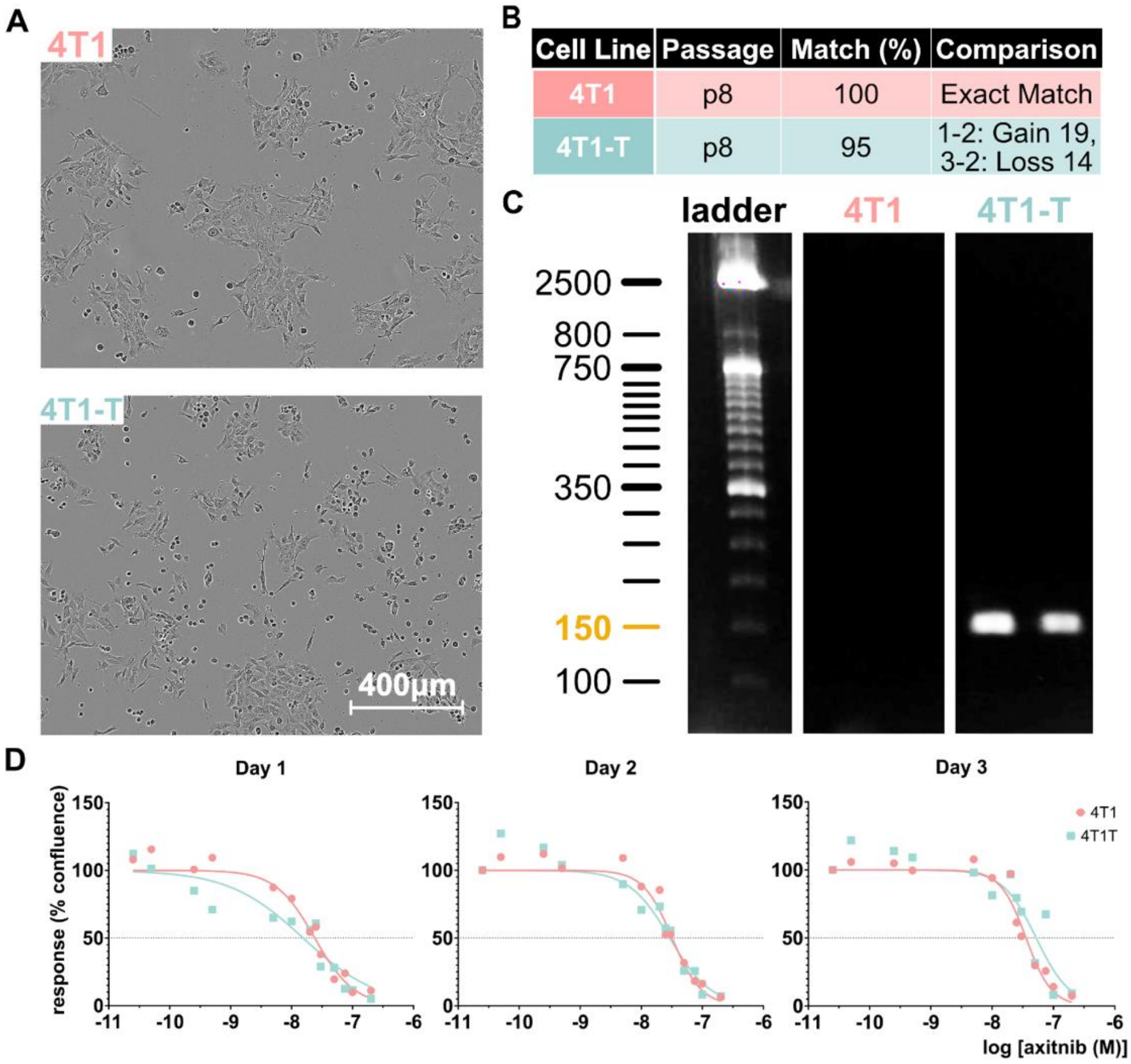

Figure S1. 4T1-parental and 4T1-T clonal characterisation. A. Brightfield captures of monolayers in 4x magnification are shown for the two cell lines. Scalebar = 400 microns. **B.** Short tandem repeats genotyping shows >95% match for both the parental and the derivative clone. **C.** Southern blotting of early and later (but no greater than 25) cell passages from each of the two clones. The R3 barcode, as reported on the original paper [1], is unique for the 4T1-T derivative clone and the expected band appears at ~150 bp. The original gel is shown in Figure S15. **D.** Dose-response axitinib curves for 1-, 2- and 3-days post-treatment at day zero for each of the clones as opposed to the log[c] of the drug concentration. IC_{50} concentrations range estimates based on the 95% profile likelihood are 0.018-0.032 µM (Day 1), 0.026-0.04

μM (Day 2) and 0.031-0.042 μM (Day 3) for the 4T1 cells, and 0.008-0.026 μM (Day 1), 0.022-0.39 μM (Day 2) and 0.36-0.08 μM (Day 3) for the 4T1-T cells, respectively.

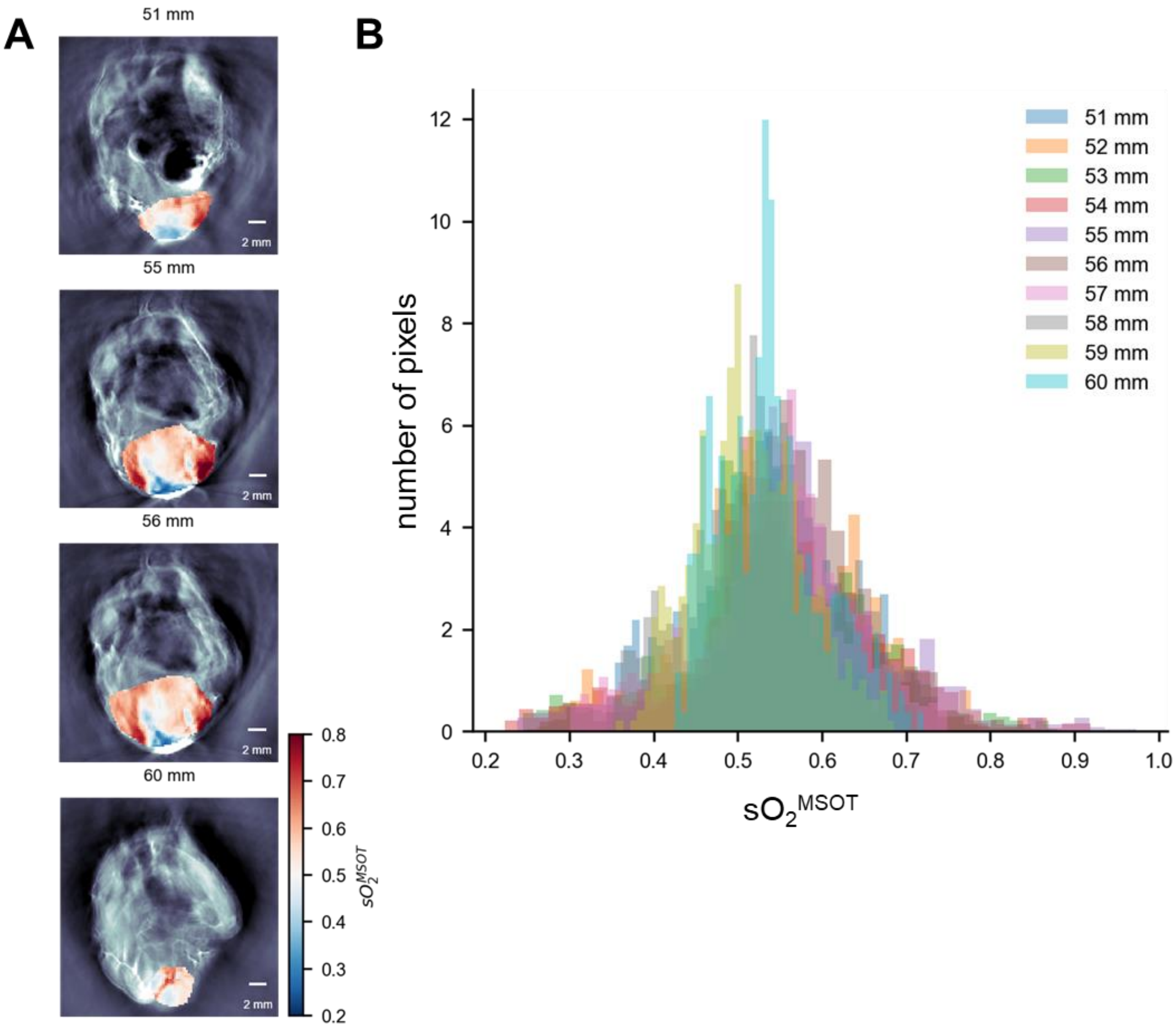

Figure S2. Photoacoustic tomography volumetric analysis. A. Indicative planar cross sections of the mouse body depicting sO_2^{MSOT} maps across the tumour mass. Scalebar is set at 2 mm. **B.** Histogram of the distribution of sO_2^{MSOT} shows oxygenation uniformity within the

different tumour regions, such that it can be assumed that whole tumour oxygenation can be represented by single central slice analysis.

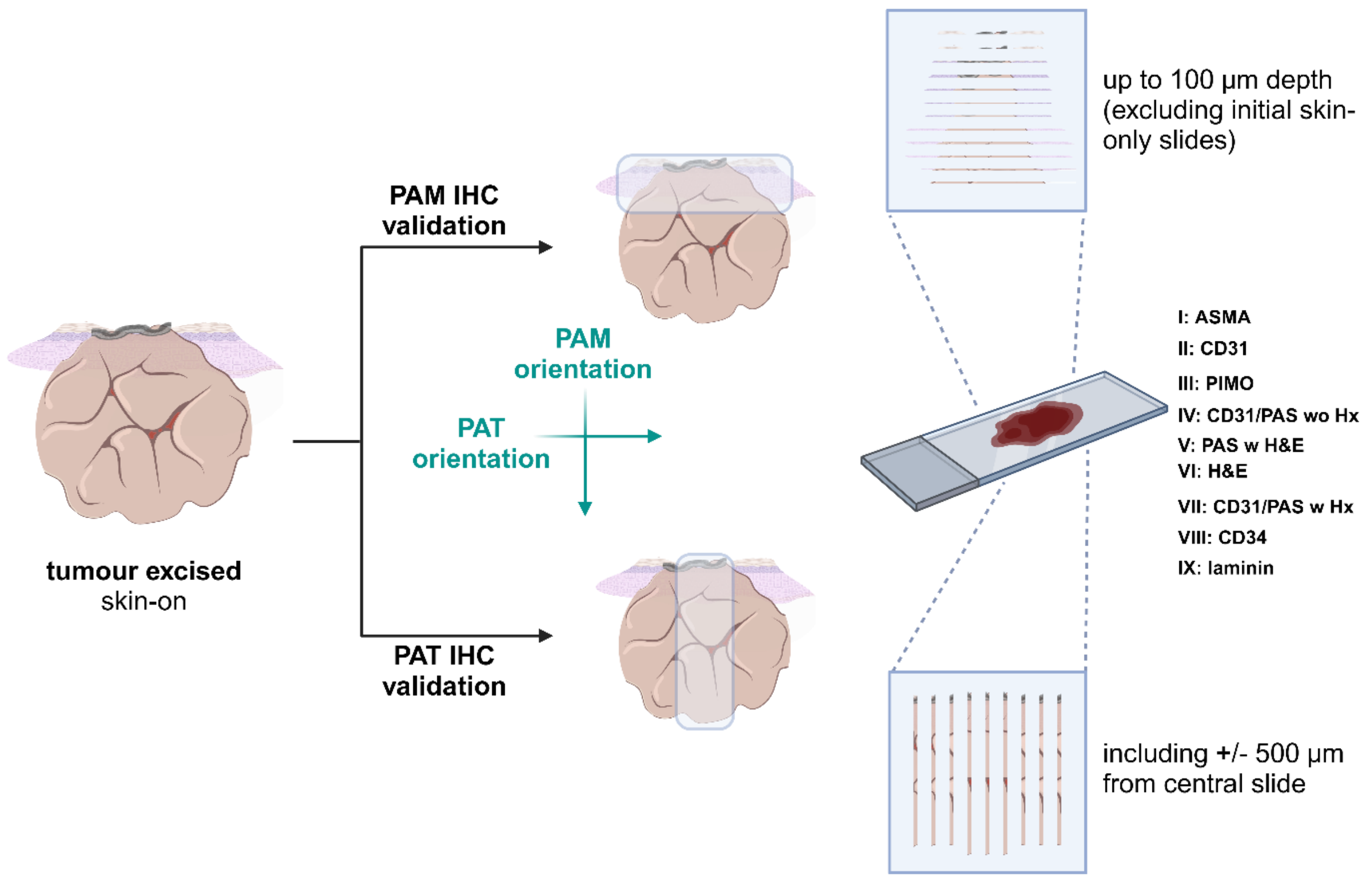

Figure S3. The immunohistochemistry (IHC) protocol. Two different sets of slides are stained following the orientation of the two different modalities; PAM represents the

mesoscopic system and reflects the tumour rim, while PAT represents the tomography system and reflects the tumour core. Created with BioRender.com

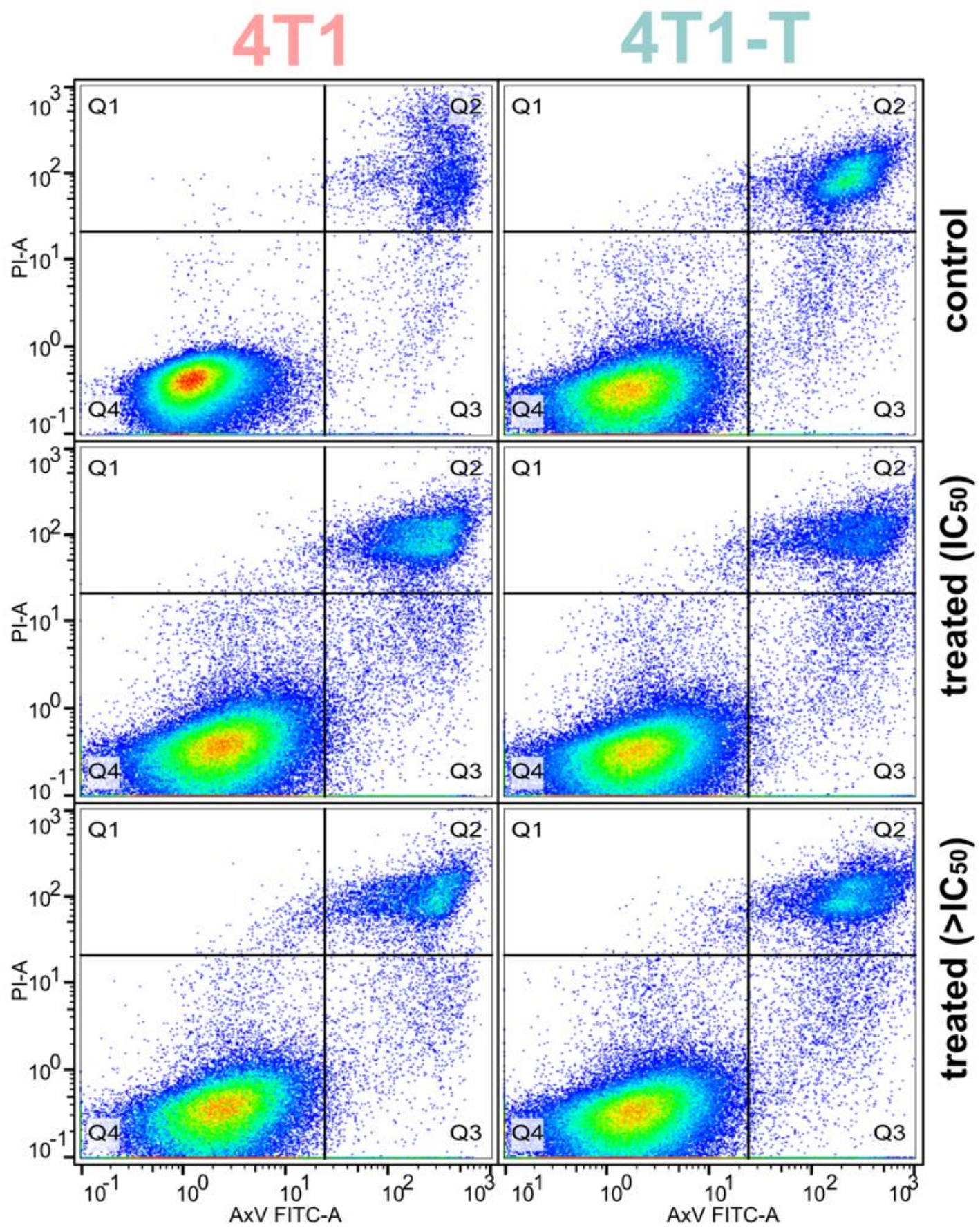

	Q1	Q2	Q3	Q4
4T1/ control	0.061	4.57	1.23	94.1
4T1/ treated (IC_{50})	0.23	9.4	3.52	86.8
4T1/ treated (>IC_{50})	0.48	8.81	3.22	87.5
4T1-T/ control	0.24	10.2	2.65	87
4T1-T/ treated (IC_{50})	0.2	5.52	3.17	91.1
4T1-T/ treated (>IC_{50})	0.28	8.39	3.14	88.2

Figure S4. Cell death estimation for the two 4T1 cell lines under different treatment conditions. Three conditions are shown from a representative experiment: no treatment (upper row), treatment within the respective IC_{50} Axitinib concentrations range (middle row), and treatment in higher than the IC_{50} Axitinib concentrations range (0.05 μM for both cell lines, lower row). The different cell population components are distributed in the four quartiles. Q1 is dead cells that are permeant to propidium iodide, Q2 is mainly necrotic cells stained for both

propidium iodide and annexin V, Q3 are apoptotic cells stained with annexin V, Q4 are the viable cells. The quartile percentage estimates are shown in the table for each condition.

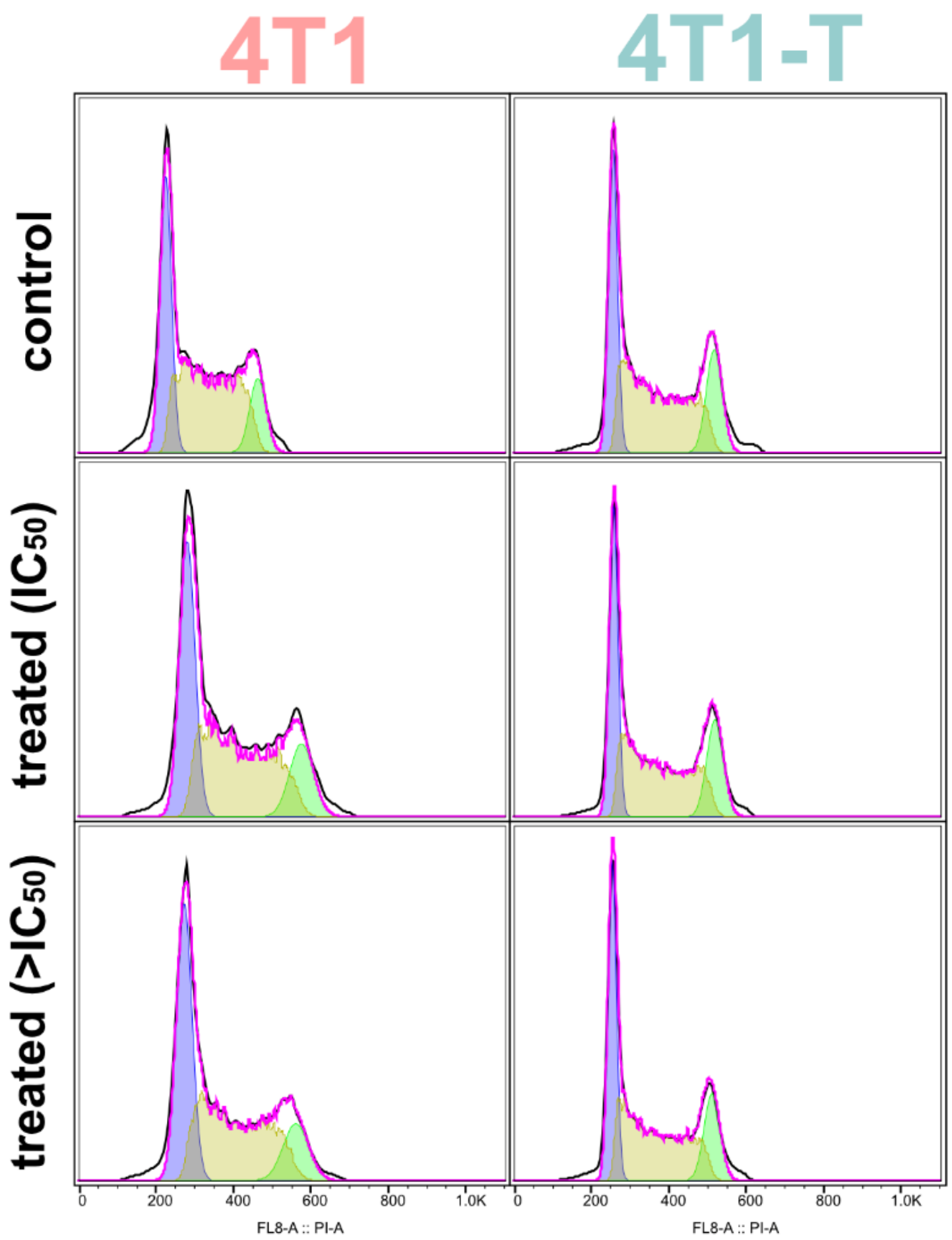

	%G1	%S	%G2
4T1/ control	31.7	51.6	11.8
4T1/ treated (IC_{50})	34.3	47.5	13.6
4T1/ treated (>IC_{50})	38	45.4	12.4
4T1-T/ control	28.3	49.4	16.5
4T1-T/ treated (IC_{50})	30.5	47.2	17.7
4T1-T/ treated (>IC_{50})	32.3	46.4	16.1

Figure S5. Cell cycle phase estimation for the two 4T1 cell lines. Three conditions are shown from a representative flow cytometry experiment: no treatment (upper row), treatment within the respective IC_{50} Axitinib concentrations range (middle row), and treatment in higher than the IC_{50} Axitinib concentrations range (0.05 μM for both cell lines, lower row). Fixed cells uptake propidium iodide staining differently based on their DNA content, so that the blue peak

refers to cells on G1 phase, the yellow peak is for cells on S phase and cells that undergo the G2/M phase are shown on the green peak. The different cell phases percentage estimates are shown in the table for each condition.

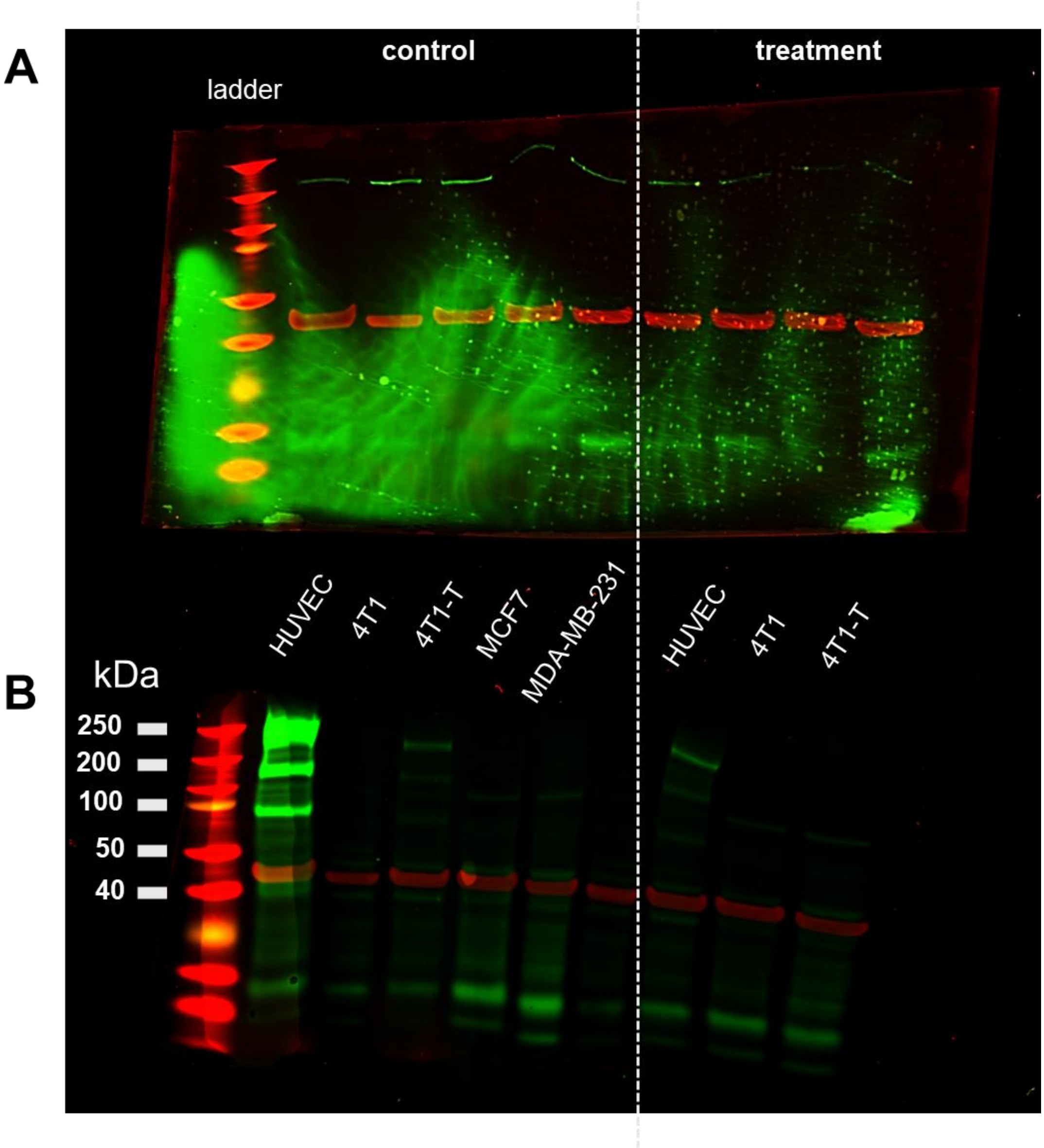

Figure S6. Original electrophoresis western blot gels. Each of the angiogenic receptors was tested on a separate gel. The endothelial HUVEC cells that have all the angiogenic receptors present were used as a reference cell line. β-Actin was used as the housekeeping

gene-coded protein. **A.** VEGFR-1 and **B.** VEGFR-2 gels are shown for control (left) and Axitinib treatment (right) conditions, respectively.

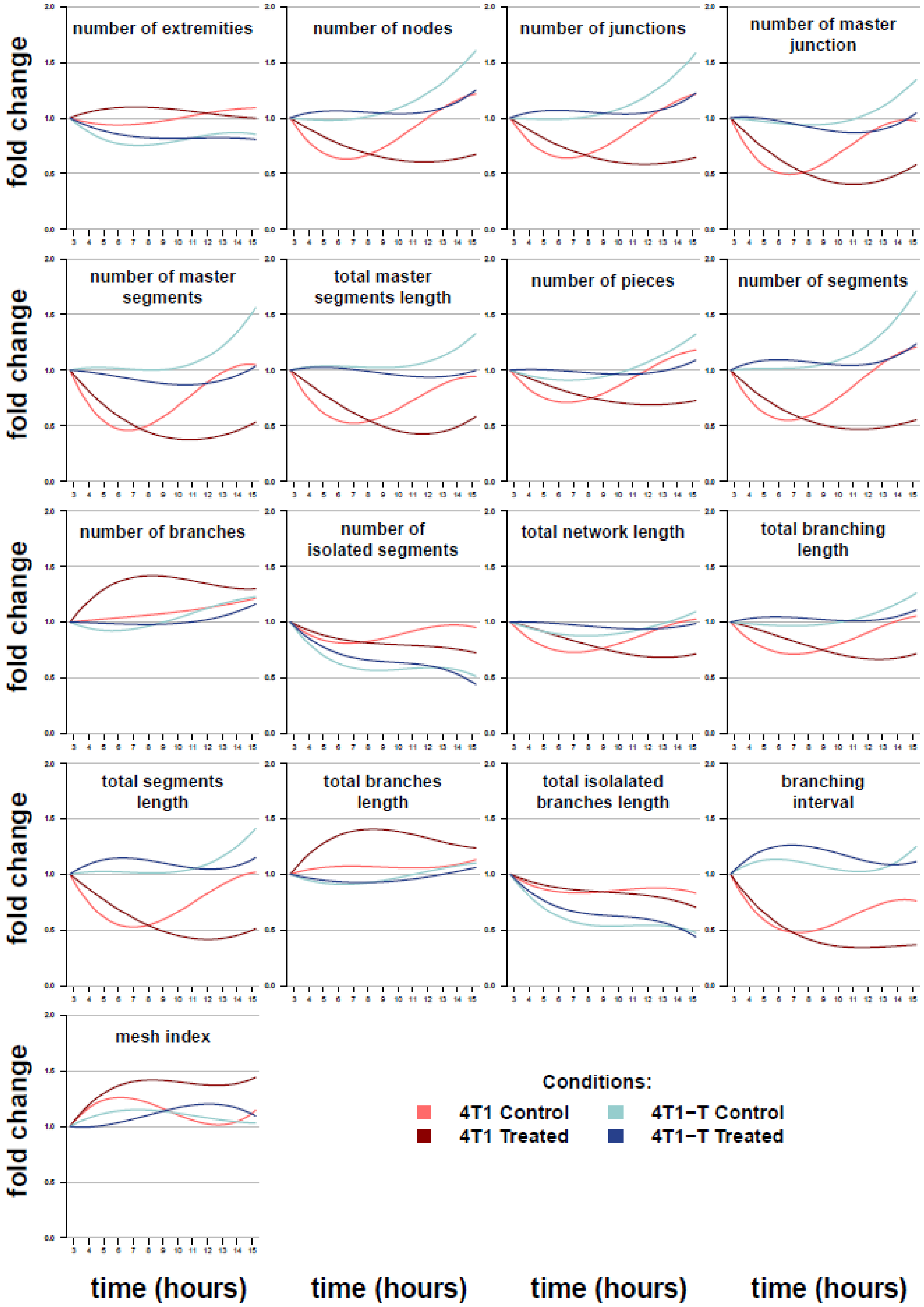

Figure S7. Vectorial objects over time of the 4T1 and 4T1-T pseudo-vascular networks with and without treatment. Normalised average object value (y-axis) versus time in hours (x-axis) is shown for the linear regression estimator.

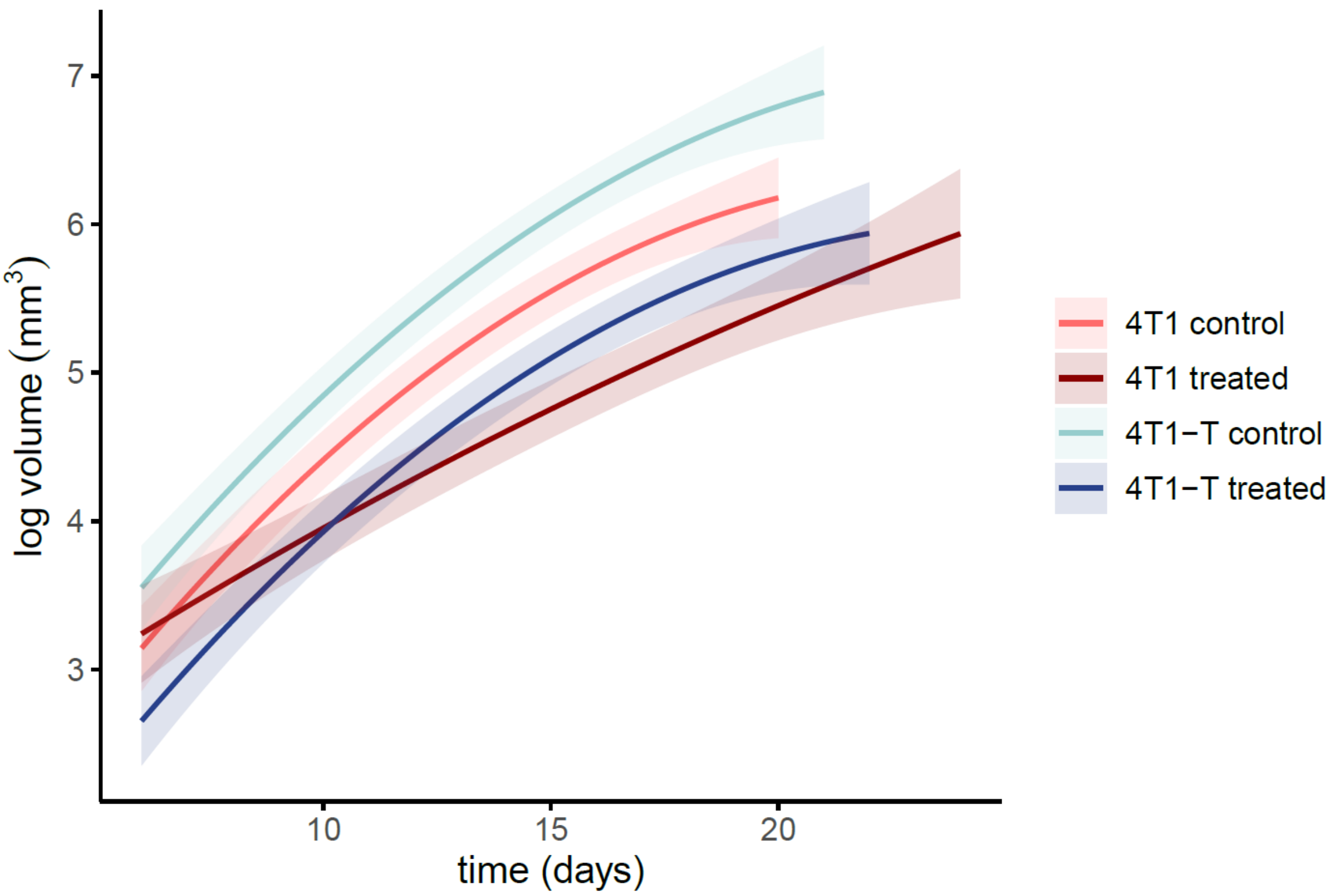

Figure S8. Tumour growth curves on natural log scale. The presence of larger tumour volumes over time in the control vs treated cohorts is a result of larger baseline tumour volumes, not because of faster tumour growth rate. At day 6 the tumour volume median predicted by the reference model (see below Supplementary Table S2) was 34.9 mm^3 for the control vs 14.2 mm^3 for the treated cohort for the 4T1-T model ($p<0.001$), while 23.2 mm^3 for the control vs 25.6 mm^3 for the treated cohort in the 4T1 model ($p=0.666$). Tumour growth rate decreases over time for every group ($p<0.001$) except for the 4T1 treated cohort ($p=0.308$). For the 4T1 model, a linear tumour growth rate was estimated equal to 3.58 mm^3 (95% CI 2.95 - 4.20 mm^3) each 10 days in the control and 1.87 mm^3 (95% CI 1.21 - 2.52 mm^3) each 10 days in the treated cohort. Moreover, the median increase of tumour volume each 10 days

(Δ_{10}) was 59.7% (95% CI_{Δ} 73.1-39.7%) lower in the treated than the Δ_{10} of the control cohort (i.e. ratio between Δ_{10} of the treated and Δ_{10} of the control cohort = 0.403). For the 4T1-T model, a linear tumour growth rate was estimated equal to 3.60 mm^3 (95% CI 3.04 - 4.16 mm^3) each 10 days in the control and 3.57 mm^3 (95% CI 2.98 - 4.16 mm^3) each 10 days in the treated cohort. Moreover, the Δ_{10} of the treated cohort was 6.0% lower (i.e. ratio between Δ_{10} of the treated and Δ_{10} of the control cohort = 0.940). However, the upper bound of the 95% CI_{Δ} showed a higher Δ_{10} of the treated cohort (lower bound of 95% CI_{Δ}: Δ_{10} was 33.6% lower in the treated than the control cohort; upper bound of 95% CI_{Δ}: Δ_{10} was 32.9% greater in the treated than the Δ_{10} of the control cohort).

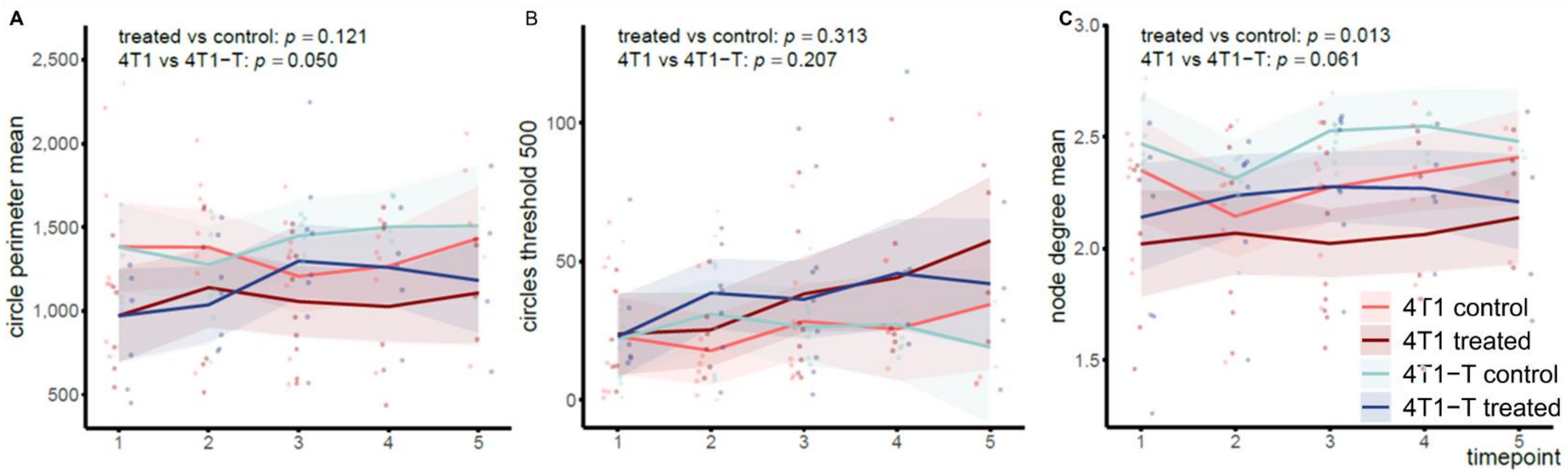

Figure S9. Photoacoustic mesoscopy graph features of the murine breast cancer models vascular architecture. A. The mean perimeter of the circles is plotted over time. The perimeter of each circle is calculated based on euclidean distances between their nodes, irrespective of the number of nodes contributing to the circle. The voxel size (20 x 20 x 4 microns) is considered. **B.** The number of circles with perimeter less or equal than 500 μm is plotted over time. The same trend was observed for cycles thresholded at 700 μm. The treatment and model effect p values are shown for each of the plots. Shaded area depicts

95% confidence intervals. **C.** The mean of the node degree, indicative of the average number of connections each node has within the vascular network, is plotted over time.

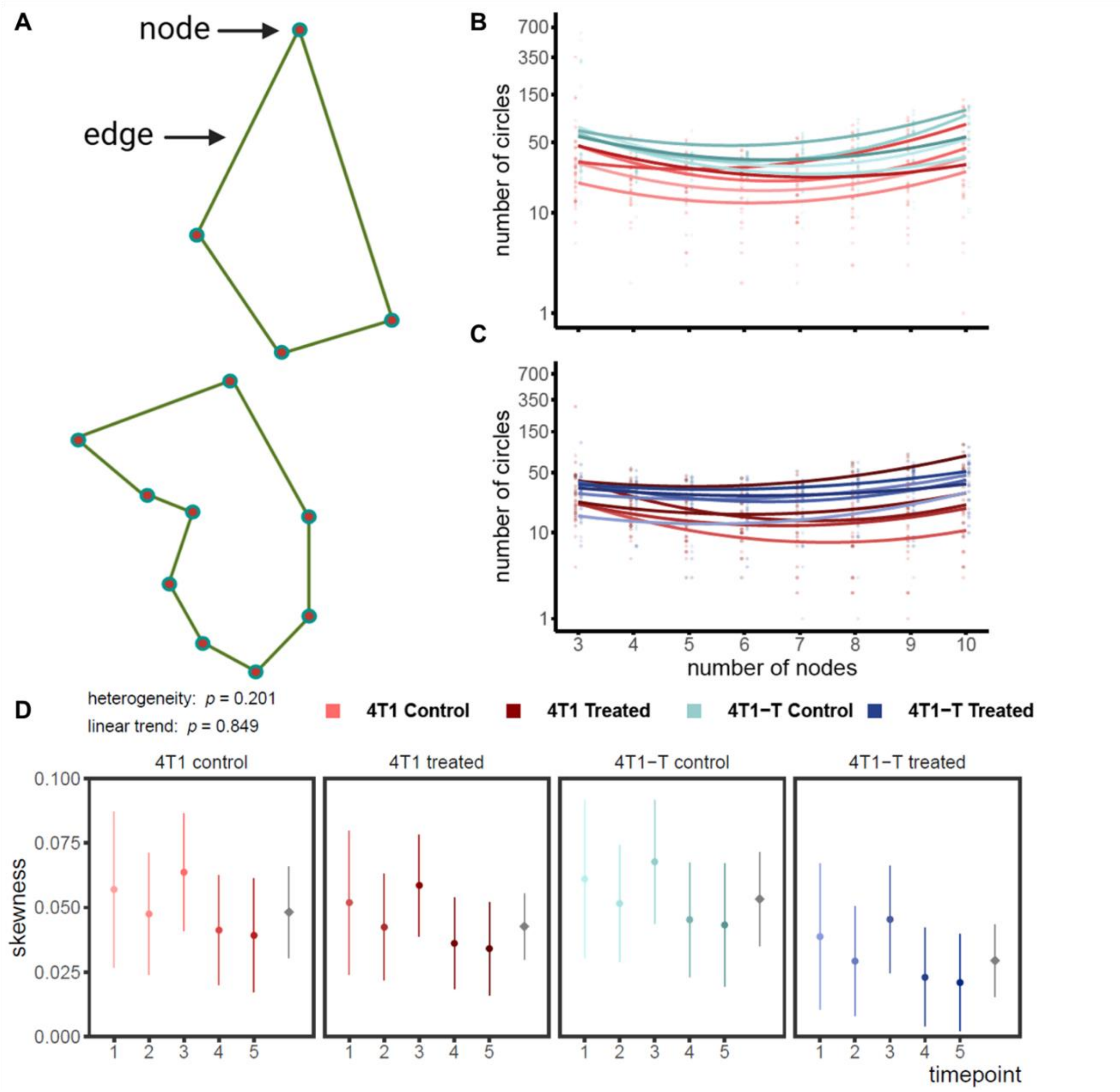

Figure S10. The complexity of the circular vessels as an estimate of vascular remodelling. A. Examples of circles within graph. A circle is defined by a minimum of 3 nodes provided that every node has at least 2 edges. Circles containing up to 10 nodes are explored. Regardless of the number of the contributing nodes, circular blood vessels may or may not have the same perimeter, meaning the sum of the edge length. **B.** For the 4T1 and 4T1-T control and **C.** the respective treated cohorts, the number of circles is plotted over the number of nodes contributing per circle for each timepoint. The darker the colour shade, the later the

timepoint for each cohort. **D.** The effect of time on the number of circles over the number of nodes is estimated by the curvature (quadratic term of the regression model). Each cohort is plotted separately. Overall curvature by cohort is shown in grey. The distribution of the nodes is positively skewed for most cohorts (convexity), towards circles of fewer (closer to 3) and more (closer to the upper end of the x axis) contributing nodes, representing coexistence of simpler and more complicated circles' morphology, respectively. P values are reported for heterogeneity and linear trend of convexity over timepoints.

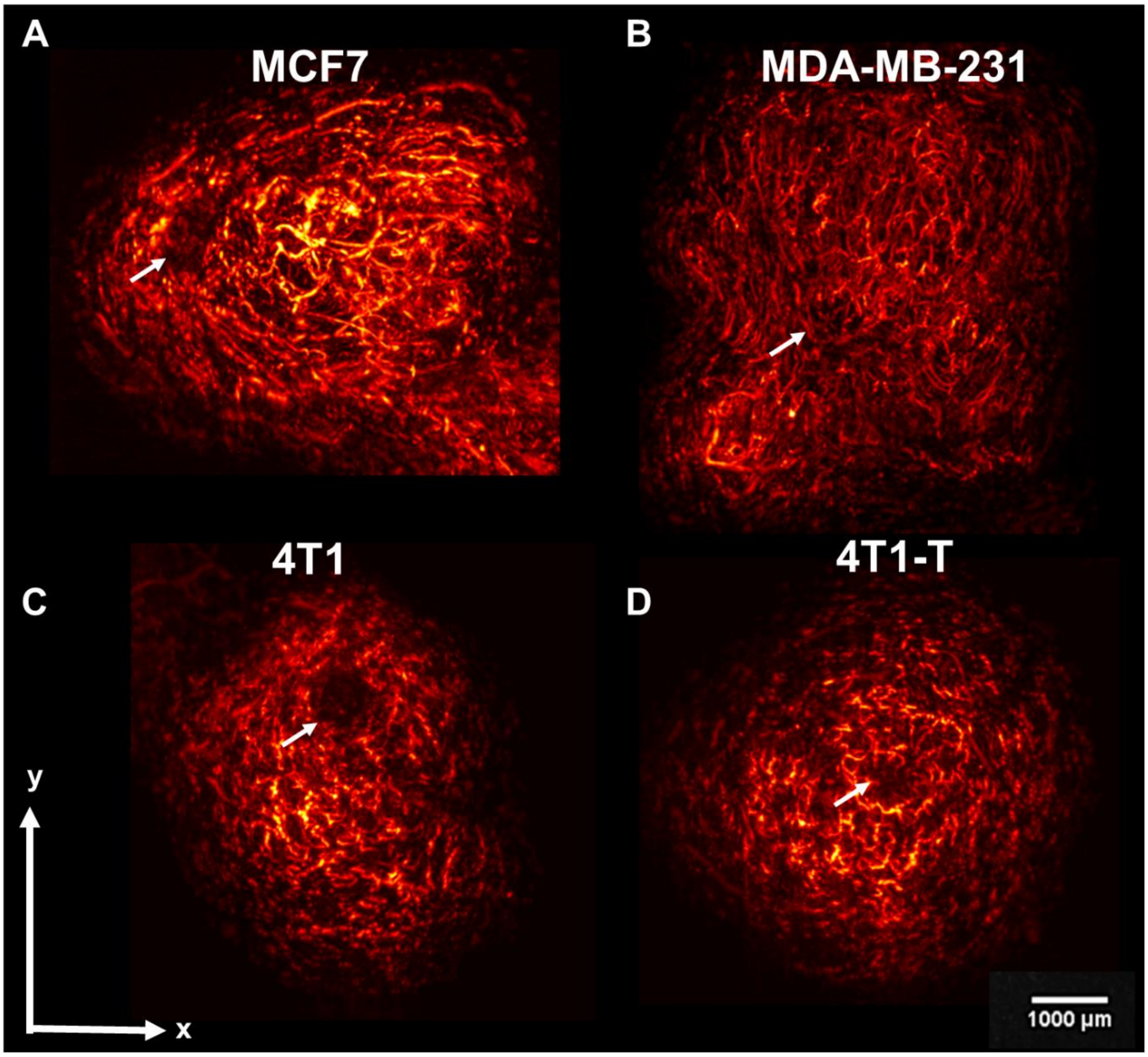

Figure S11. Photoacoustic mesoscopy pre-processed vascular networks. A. MCF7, **B.** MDA-MB-231, **C.** 4T1 and **D.** 4T1-T orthotopic tumour examples are shown for intermediate timepoints. Arrows indicate murine mammary papilla light absorbance due to skin pigmentation. Notice that the MCF7 vascular network is less tortuous than those of the other tumour models shown.

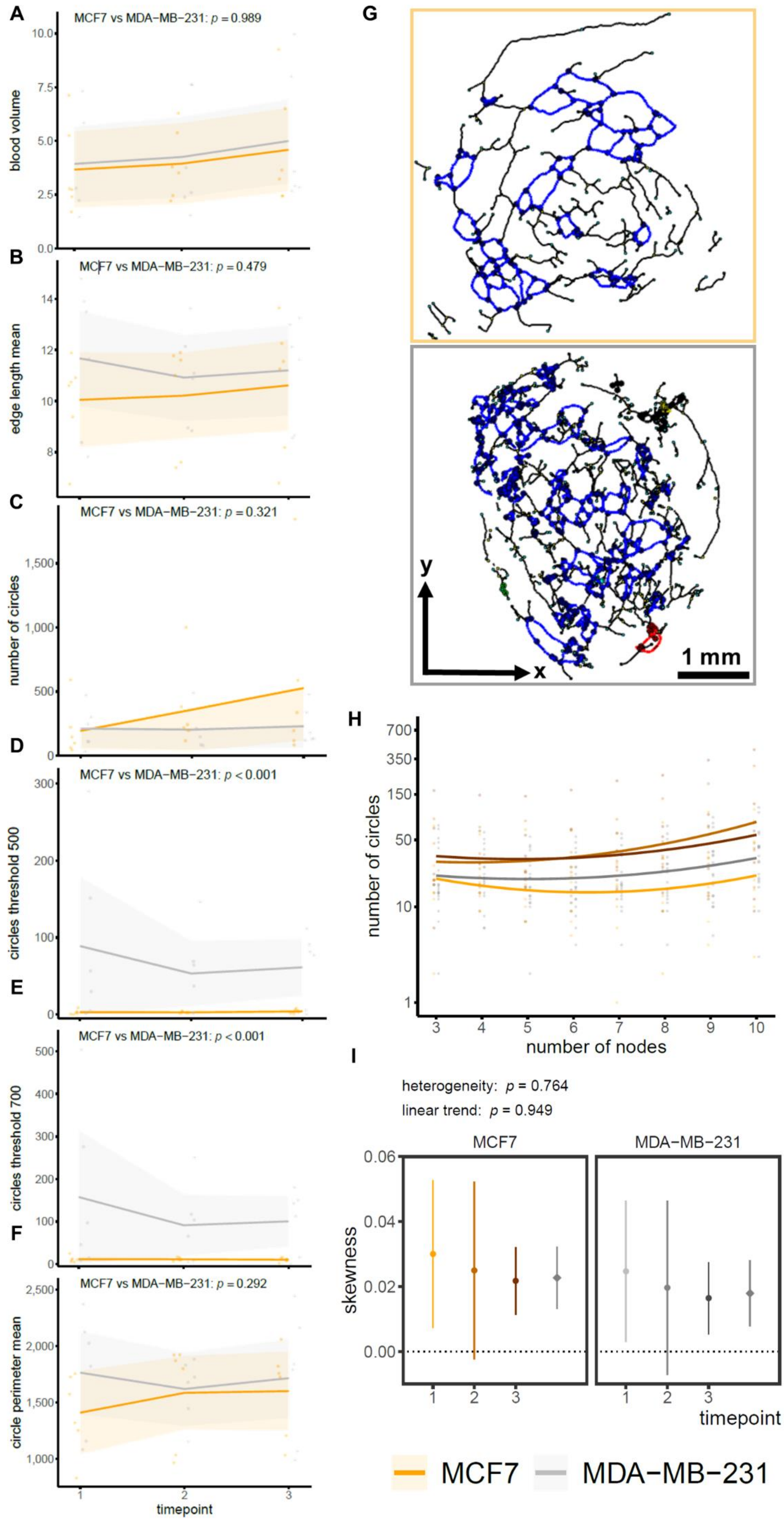

A
MCF7 vs MDA−MB−231: p = 0.989
blood volume
B
MCF7 vs MDA−MB−231: p = 0.479
edge length mean
C
MCF7 vs MDA−MB−231: p = 0.321
number of circles
D
MCF7 vs MDA−MB−231: p < 0.001
circles threshold 500
E
MCF7 vs MDA−MB−231: p < 0.001
circles threshold 700
F
MCF7 vs MDA−MB−231: p = 0.292
circle perimeter mean
timepoint
G
y
x
1 mm
H
number of circles
number of nodes
I
heterogeneity: p = 0.764
linear trend: p = 0.949
MCF7
MDA−MB−231
skewness
timepoint
MCF7
MDA−MB−231

Figure S12. Photoacoustic mesoscopy graph analysis of the human breast cancer models. The blood volume (**A**), the mean vessel length (**B**), the total number of circles (**C**), the number of the circles with perimeter less or equal than 500 microns (**D**) and 700 microns (**E**) and the mean circle perimeter (**F**) are plotted over time for the MCF7 and the MDA-MB-231 cohorts. The model effect p values are shown for each of the plots. Shaded area depicts 95% confidence intervals. **G.** Graphs are shown for MCF7 and MDA-MB-231 representative datasets for the earliest timepoint. The human breast cancer models are size-matched. Circles originating from the same connected component are depicted in the same colour. The xy plane is shown. Scalebar is set at 2 mm. **H.** The number of circles is plotted over the number of nodes contributing per circle for each timepoint. **I.** The effect of time on the number of circles over the number of nodes is estimated by the convexity. Each cohort is plotted separately. P values are reported for heterogeneity and linear trend of convexity over timepoints.

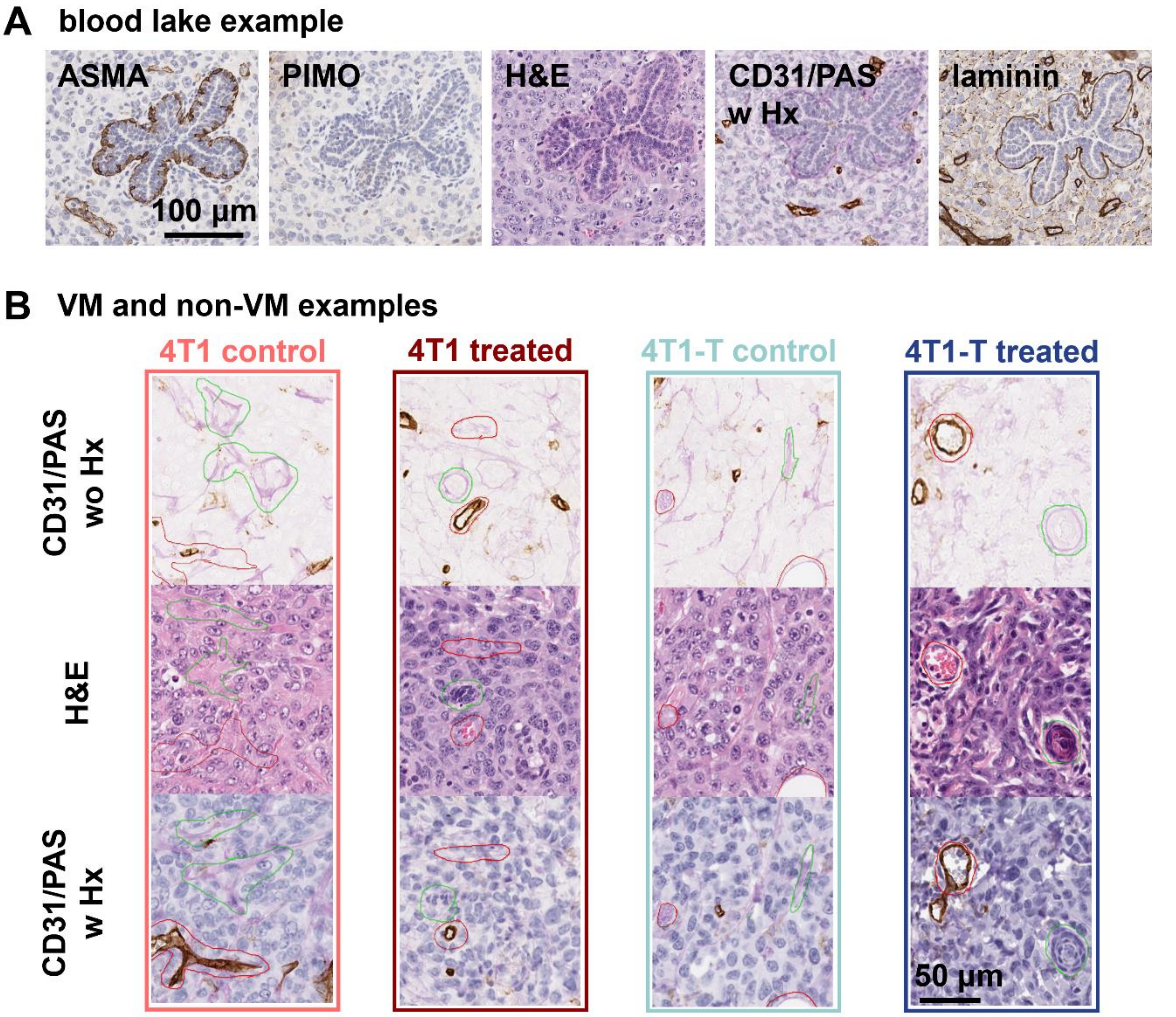
A
blood lake example
ASMA
PIMO
H&E
CD31/PAS w Hx
laminin
100 µm
B
VM and non-VM examples
4T1 control
4T1 treated
4T1-T control
4T1-T treated
CD31/PAS wo Hx
H&E
CD31/PAS w Hx
50 µm

Figure S13. IHC analysis of the murine breast cancer models accounting for the tumour rim and core. The number of VM annotations, meaning CD31$^-$/PAS$^+$ areas in the viable tumour regions are depicted for the tumour rim (**A**) and the tumour core (**B**) over time for each of the cohorts. Annotations in red and green depict non-VM and VM examples, respectively.

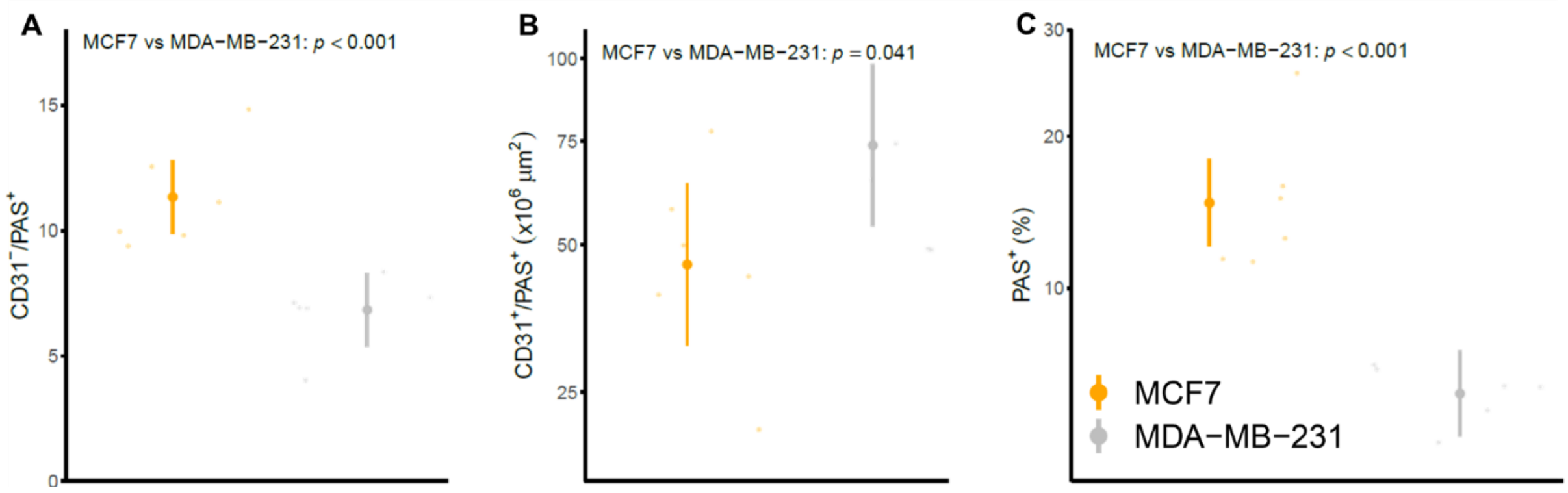

Figure S14. Histological and IHC analysis of the human breast cancer models. The CD31$^-$/PAS$^+$VM annotations (**A**), the CD31$^+$/ PAS$^+$ non-VM annotations (**B**), and the percentage of PAS$^+$ in the viable tissue (**C**) are depicted for each of the cohorts at endpoint. Model effect p values are shown for each of the plots. Segments depict 95% confidence intervals.

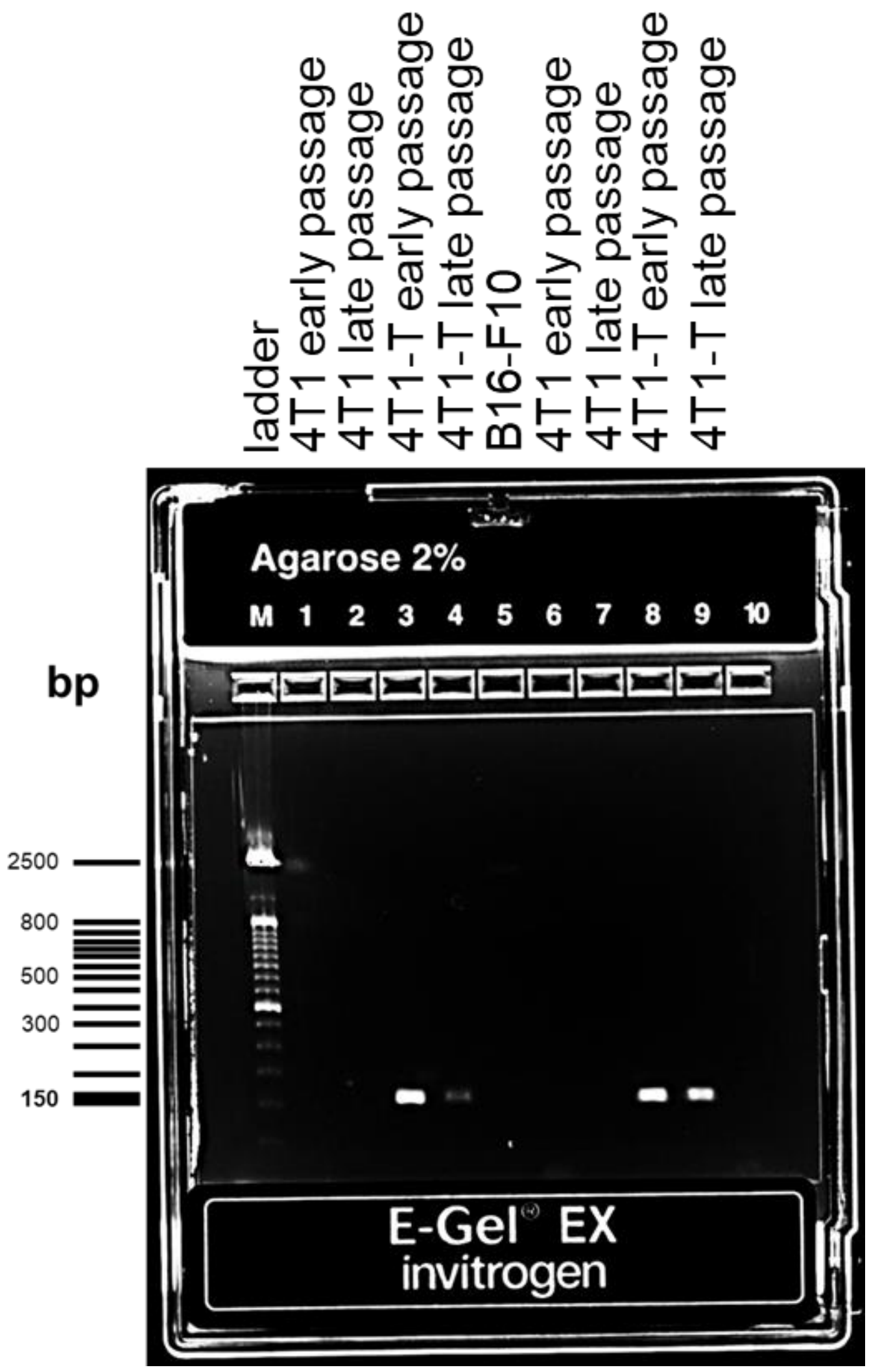

Figure S15. Original electrophoresis southern blot gel. Both early and late cell passages (but not greater than 25) were tested for 4T1 and 4T1-T cell lines. The melanoma B16-F10 murine cell line was used as a negative control. The R3 barcode, as reported on the original paper [1], is unique for the 4T1-T derivative clone and is expected at the 150 bp band.

Supplementary Methods: Statistical Analysis

For tumour growth data (Figure 2C and Supplementary Figure S8), the dependent variable was analysed on natural log scale and the time predictor was considered as quadratic polynomial. Generalised least squares were used to fit linear fixed-effects models (LFMs) with unequal variances. Residuals assumptions of linear models were checked using graphical methods (i.e. quantile-quantile plots, scatter plots and histograms), descriptive statistics (i.e. mean and standard deviation) and formal methods (i.e. Shapiro-Wilk test, Bartlett's test and likelihood ratio test with restricted maximum likelihood as estimator). Generalised linear mixed-effect models (GLMMs) were used to detect and estimate the dependency of circles count on number of nodes (Supplementary Figure S10B-C, convex curves). The negative binomial distribution, the dependence of the dispersion parameter on timepoints and a random intercept for each mouse subject were used to model the random component of the generalised linear mixed-effect model (LMM) reference statistical model (RSM). Number of nodes was considered as quadratic polynomial, timepoints as categorical variable and their interaction were used as predictors.

Sensitivity analyses were performed to identify the effect of outliers on conclusions for each RSM. After outliers' identification, the RSM was fitted on the dataset excluding outliers. The analysis without outliers confirmed the results of the primary analysis. The following alternative statistical models were examined: a) the reference statistical model with other covariance structures of random effects and residuals; b) data transformation on $\log_e$ (x+1), square root and rank scales; c) time as linear and quadratic polynomial; d) constant residuals variance (homoscedasticity) and constant dispersion parameter in the negative binomial distribution; e) the reference statistical model with an interaction term between time, treatment and model. Alternative data models confirmed the results of the primary analysis. The likelihood ratio test with maximum likelihood as estimator was used to test primary hypotheses. Simultaneous z-tests for general linear hypotheses were used as post-hoc tests. The Holm's sequential

Bonferroni procedure [2][2] was used to compute adjusted p-values on primary features. An adjusted p-value less than 0.01 was considered statistically significant.

All statistical analysis was performed using R Statistical Software (v4.4.1; R Core Team 2024). LMMs and LFMs were analysed using the *nlme* R package (v3.1.164) [3]. Fitting of LMMs and LFMs using generalised least squares were performed using *lme* and *gls* functions, respectively. GLMMs were analysed using the glmmTMB R package (v1.1.10) [4]. Quantile residuals for fitted GLMMs were estimated, visually and formally checked using the DHARMa R package (v0.4.7) [5]. Simultaneous z-tests for general linear hypotheses with the single-step method and Wald tests were calculated using the *glht* function of the *multcomp* R package (v1.4.26) [6]. Outliers were identified using the *robustbase* R package (v0.99.4) [6]. Data visualisation was performed using base R and the *ggplot2* R package (v3.5.1) [7].

Tables

Table S1. A list of all mice enrolled in the study. The main procedures are included. Imaging timepoints are shown. Asterisk indicates gas challenge during PAT imaging. Endpoint is shown in red in case terminal experiment was not successfully completed. Two planes at histology refer to PAM and PAT imaging orientation.

		**Imaging timepoints**									
**Mouse ID**	**Cohort**	**1**	**2**	**3**	**4**	**5**	**6**	**7**	**8**	**Histology**	**Comments**
**MEO01**	4T1/ control						*			2 planes	ulceration
**MEO02**	4T1-T/ control						*			2 planes	ulceration
**MEO03**	4T1/ control					*				2 planes	health concerns
**MEO04**	4T1-T/ control						*			2 planes	ulceration
**MEO05**	4T1/ control					*				2 planes	
**MEO06**	4T1-T/ control			*						2 planes	ulceration, growth outlier
**MEO07**	4T1/ treated							*		2 planes	ulceration

**MEO08**	4T1-T/ treated							*		2 planes	ulceration
**MEO09**	4T1/ control						*			2 planes	
**MEO10**	4T1-T/ control					*				2 planes	ulceration
**MEO11**	4T1/ treated								*	2 planes	
**MEO12**	4T1-T/ treated						*			2 planes	
**MEO13**	4T1/ control					*				2 planes	ulceration
**MEO14**	4T1-T/ control				*					2 planes	ulceration
**MEO15**	4T1/ treated						*			2 planes	ulceration
**MEO16**	4T1-T/ treated						*			2 planes	ulceration
**MEO17**	4T1/ control						*			2 planes	
**MEO18**	4T1-T/ control					*				2 planes	ulceration
**MEO19**	4T1/ treated						*			2 planes	ulceration
**MEO20**	4T1-T/ treated						*			2 planes	ulceration
**MEO21**	4T1/ control		*							2 planes	MRI imaging
**MEO22**	4T1-T/ control		*							2 planes	MRI imaging
**MEO23**	4T1/ control		*							2 planes	MRI imaging
**MEO24**	4T1-T/ control		*							2 planes	MRI imaging
**MEO25**	4T1/ control			*						2 planes	
**MEO26**	4T1/ control			*						2 planes	
**MEO27**	4T1/ control				*					2 planes	
**MEO28**	4T1/ control			*						2 planes	
**MEO29**	4T1/ control				*					2 planes	
**MEO30**	4T1-T/ control				*					2 planes	
**MEO31**	4T1-T/ control				*					2 planes	
**MEO32**	4T1-T/ control			*						2 planes	
**MEO33**	4T1-T/ control			*						2 planes	
**MEO34**	4T1-T/ control			*						2 planes	
**MEO35**	4T1/ treated				*					2 planes	only PAT imaging/ growth outlier

**MEO36**	4T1/ treated				*					2 planes	only PAT imaging/ growth outlier
**MEO37**	4T1/ treated				*					2 planes	only PAT imaging/ growth outlier
**MEO38**	4T1/ treated				*					2 planes	only PAT imaging/ growth outlier
**MEO39**	4T1/ treated				*					2 planes	imaging/ growth outlier
**MEO40**	4T1-T/ treated				*					2 planes	only PAT imaging/ growth outlier
**MEO41**	4T1-T/ treated				*					2 planes	only PAT imaging/ growth outlier
**MEO42**	4T1-T/ treated				*					2 planes	only PAT imaging/ growth outlier
**MEO43**	4T1-T/ treated				*					2 planes	only PAT imaging/ growth outlier
**MEO44**	4T1-T/ treated				*					2 planes	only PAT imaging/ growth outlier
**MEO45**	4T1/ treated			*						2 planes	
**MEO46**	4T1/ treated			*						2 planes	
**MEO47**	4T1/ treated				*					2 planes	
**MEO48**	4T1/ treated				*					2 planes	health concerns
**MEO49**	4T1/ treated			*						2 planes	
**MEO50**	4T1-T/ treated			*						2 planes	
**MEO51**	4T1-T/ treated				*					2 planes	
**MEO52**	4T1-T/ treated			*						2 planes	
**MEO53**	4T1-T/ treated				*					2 planes	second tumour appear
**MEO54**	4T1-T/ treated			*						2 planes	
**MEO55**	4T1/ control			*						2 planes	
**MEO56**	4T1/ control			*						2 planes	
**MEO57**	4T1/ control				*					2 planes	
**MEO58**	4T1/ control				*					2 planes	
**MEO59**	4T1/ control			*						2 planes	
**MEO60**	4T1-T/ control				*					2 planes	
**MEO61**	4T1-T/ control				*					2 planes	
**MEO62**	4T1-T/ control			*						2 planes	nodular tumour
**MEO63**	4T1-T/ control			*						2 planes	

**MEO64**	4T1-T/ control			*						2 planes	ulceration
**TLL01**	MDA-MB-231									central plane	only PAM imaging
**TLL02**	MCF7									central plane	only PAM imaging
**TLL03**	MCF7									central plane	only PAM imaging
**TLL04**	MDA-MB-231									central plane	only PAM imaging
**TLL05**	MDA-MB-231									central plane	only PAM imaging
**TLL06**	MDA-MB-231									central plane	only PAM imaging
**TLL07**	MDA-MB-231									central plane	only PAM imaging
**TLL08**	MDA-MB-231									central plane	only PAM imaging
**TLL09**	MCF7									central plane	only PAM imaging
**TLL10**	MCF7									central plane	only PAM imaging
**TLL11**	MCF7									central plane	only PAM imaging
**TLL12**	MCF7									central plane	only PAM imaging

Table S2. A list of all the features extracted. The variables are categorised per modality. Statistical tests and the respective p values are denoted.

Modality	Feature	In text	Category	Comparison	Statistical test	p value
**tumour growth**	tumour volume (volume)	Figures 2C, S8	exploratory	interaction treatment effect and model	conditional F-test	***0.009***
				treatment effect in model 4T1		***<0.001***
				treatment effect in model 4T1-T		0.876
				model effect		0.571
**PAT**	saturation of oxygen (sO_2)	Figures 2D, 2F, S2	exploratory	treated vs control	likelihood ratio test	*0.046*
				4T1 vs 4T1-T		0.387
	standard deviation of sO_2 (sO_2 std)	Figure 3A	primary	treated vs control	likelihood ratio test	***<0.001***
				4T1 vs 4T1-T		***0.003***
	sO_2 std for tumour rim	Figure 3B	exploratory	treated vs control	likelihood ratio test	***<0.001***
				4T1 vs 4T1-T		***0.002***
	sO_2 std for tumour core	Figure 3B	exploratory	treated vs control	likelihood ratio test	***0.010***
				4T1 vs 4T1-T		***<0.001***
	total haemoglobin (tHb)	Figure 2E	exploratory	treated vs control	likelihood ratio test	*0.022*
				4T1 vs 4T1-T		0.567
	tumour delta sO_2 (ΔsO_2)	Figures 3C, 3E	exploratory	treated vs control	likelihood ratio test	0.072
				4T1 vs 4T1-T		***0.001***
	tumour responding fraction (RF)	Figures 3D, 3F	exploratory	treated vs control	likelihood ratio test	0.524
				4T1 vs 4T1-T		*0.020*
**PAM**	number of circles	Figure 4H	primary	treated vs control	likelihood ratio test	***0.007***
				4T1 vs 4T1-T		***0.007***
		Figure S12C	exploratory	MCF7 vs MDA-MB-231		0.321
		Figures S9B, S9C		treatment and 4T1/4T1-T model effect	Wald test	*0.013*

			treatment in 4T1		***0.002***
			treatment in 4T1-T		*0.014*
number of circles/ ‘bathtub’ coefficient	Figures S10B, S10C	exploratory	heterogeneity in time effect in 4T1/4T1-T models	Wald test	0.201
			linearity in time effect in 4T1/4T1-T models		0.849
	Figures S12H, S12I		heterogeneity in time effect in MCF7/MDA-MB-231 models		0.764
			linearity in time effect in MCF7/MDA-MB-231 models		0.949
circles threshold 500 µm	Figure S9C	exploratory	treated vs control	likelihood ratio test	0.313
			4T1 vs 4T1-T		0.207
	Figure S12D		MCF7 vs MDA-MB-231		***<0.001***
circles threshold 700 µm	-	exploratory	treated vs control	likelihood ratio test	0.430
			4T1 vs 4T1-T		0.119
	Figure S12E		MCF7 vs MDA-MB-231		***<0.001***
circle perimeter	Figure S9A	exploratory	treated vs control	likelihood ratio test	0.121
			4T1 vs 4T1-T		***0.050***
	Figure S12F		MCF7 vs MDA-MB-231		0.292
total network length (blood volume)	Figure 4G	exploratory	treated vs control	likelihood ratio test	***<0.001***
			4T1 vs 4T1-T		0.314

		Figure S12A		MCF7 vs MDA-MB-231		0.989
	mean edge length	Figure 4I	exploratory	treated vs control	likelihood ratio test	0.150
				4T1 vs 4T1-T		0.059
		Figure S12B		MCF7 vs MDA-MB-231		0.479
	mean node degree	Figure S9B	exploratory	treated vs control	likelihood ratio test	*0.013*
				4T1 vs 4T1-T		0.061
		-		MCF7 vs MDA-MB-231		0.151
**histology**	$CD31^+/ASMA^+$	Figure 5C	exploratory	PAM: treatment and 4T1/4T1-T model effect	likelihood ratio test	*0.044*
				PAM: treatment in 4T1		0.118
				PAM: treatment in 4T1-T		***0.008***
				PAM: 4T1 vs 4T1-T		0.405
				PAT: treatment and 4T1/4T1-T model effect		***<0.001***
				PAT: treatment in 4T1		***<0.001***
				PAT: treatment in 4T1-T		*0.012*
				PAT: 4T1 vs 4T1-T		0.131
	pimonidazole (hypoxia)	Figure 5D	exploratory	PAM: treated vs control	likelihood ratio test	*0.028*
				PAM: 4T1 vs 4T1-T		0.545

			PAT: treated vs control		0.177
			PAT: 4T1 vs 4T1-T		0.293
$CD31^{-}/PAS^{+}$	Figure 5E	exploratory	PAM: treated vs control	likelihood ratio test	*0.038*
			PAM: 4T1 vs 4T1-T		0.852
			PAT: treated vs control		0.162
			PAT: 4T1 vs 4T1-T		*0.027*
	Figure S14A		MCF7 vs MDA-MB-231		***<0.001***
$CD31^{+}/PAS^{+}$	Figure 5F	exploratory	PAM: treated vs control	likelihood ratio test	0.100
			PAM: 4T1 vs 4T1-T		0.105
			PAT: treated vs control		***0.008***
			PAT: 4T1 vs 4T1-T		***<0.001***
	Figure S14B		MCF7 vs MDA-MB-231		*0.041*
PAS^{+}	-	exploratory	PAM: treatment and 4T1/4T1-T model effect	likelihood ratio test	*0.042*
			PAM: treatment in 4T1		***<0.001***
			PAM: treatment in 4T1-T		***<0.001***
			PAM: 4T1 vs 4T1-T		0.969
			PAT: treated vs control		***<0.001***
			PAT: 4T1 vs 4T1-T		***<0.001***

		Figure S14C		MCF7 vs MDA-MB-231		***<0.001***
	laminin	Figure 5G	exploratory	PAM: treated vs control	likelihood ratio test	***<0.001***
				PAM: 4T1 vs 4T1-T		0.846
				PAT: treatment and 4T1/4T1-T model effect		***<0.001***
				PAT: treatment in 4T1		0.461
				PAT: treatment in 4T1-T		***<0.001***
				PAT: 4T1 vs 4T1-T		*0.044*

References

1. Wagenblast E, Soto M, Gutiérrez-Ángel S, Hartl CA, Gable AL, Maceli AR, et al. A model of breast cancer heterogeneity reveals vascular mimicry as a driver of metastasis. Nature. 2015; 520: 358-62.
2. Holm S. A Simple Sequentially Rejective Multiple Test Procedure. Scandinavian Journal of Statistics. 1979; 6: 65-70.
3. Pinheiro JC, Bates DM. nlme: Linear and Nonlinear Mixed Effects Models: R package version 3.1-168; 2025.
4. Brooks ME, Kristensen K, Benthem KJv, Magnusson A, Berg CW, Nielsen A, et al. glmmTMB Balances Speed and Flexibility Among Packages for Zero-inflated Generalized Linear Mixed Modeling. R Journal. 2017; 9: 378.
5. Florian H. DHARMa: Residual Diagnostics for Hierarchical (Multi-Level / Mixed) Regression Models. 2024. p. R package version 0.4.7.
6. Maechler M, Rousseeuw P, Croux C, Todorov V, Ruckstuhl A, Salibian-Barrera M, et al. robustbase: Basic Robust Statistics. R package version 0.99-4-1. 2024.
7. Wickham H. ggplot2: Elegant Graphics for Data Analysis: Springer-Verlag New York; 2016.
